# Recent Progress in on‐Demand Transfer‐Enabled Integration of Wavelength‐Scale Light Sources

**DOI:** 10.1002/nap2.70033

**Published:** 2026-02-21

**Authors:** Hyundong Kim, Dongmin Shin, Gon Young Bae, Gil‐Woo Lee, Hae Young Jung, Jae‐Pil So, Myung‐Ki Kim, You‐Shin No

**Affiliations:** ^1^ Department of Physics Konkuk University Seoul Republic of Korea; ^2^ KU‐KIST Graduate School of Converging Science and Technology Korea University Seoul Republic of Korea; ^3^ Department of Physics Soongsil University Seoul Republic of Korea; ^4^ Department of Integrative Energy Engineering Korea University Seoul Republic of Korea

**Keywords:** heterogeneous integration, micro/nanolaser integration, micro‐transfer‐printing, on‐chip photonic integration, PDMS stamp, quantum light sources

## Abstract

The growing demand for massive and high‐speed data processing within compact photonic circuits has highlighted a critical challenge: the efficient integration of high‐quality ultrasmall light sources and emitters onto next‐generation integration platforms. Despite notable advancements achieved through conventional and cutting‐edge strategies, integration technologies utilizing the micro‐transfer‐printing technique—employing microstructured polymeric stamps, such as polydimethylsiloxane (PDMS)—have garnered considerable attention. This innovative approach facilitates heterogeneous integration by enabling the deterministic placement of micro‐ and nanoscale optical structures and materials with sub‐micrometer alignment onto diverse photonic integration platforms. This review paper presents recent developments in the micro‐transfer‐enabled integration of light sources across four representative categories of devices and materials: microdisk and microring cavity lasers, photonic crystal nanobeam lasers, semiconductor nanowire lasers and LEDs, and quantum light sources based on semiconductor quantum dots and localized emitters in two‐dimensional materials. For each category of light source integration, we analyze the application of micro‐transfer‐printing in relation to the overall integration configuration, the desired optical properties, device performance optimization, and resolution of challenges and limitations encountered in previous methodologies. Collectively, these demonstrations position PDMS‐assisted micro‐transfer‐printing not merely as a fabrication technique but as an innovative integration paradigm that connects diverse material systems and device architectures.

## Graphical abstract

A novel integration technology using micro‐transfer‐printing, demonstrating individually and selectively addressable pick‐up of a microlaser on a growth wafer and on‐demand integration at a predetermined location on the compact PIC chip.

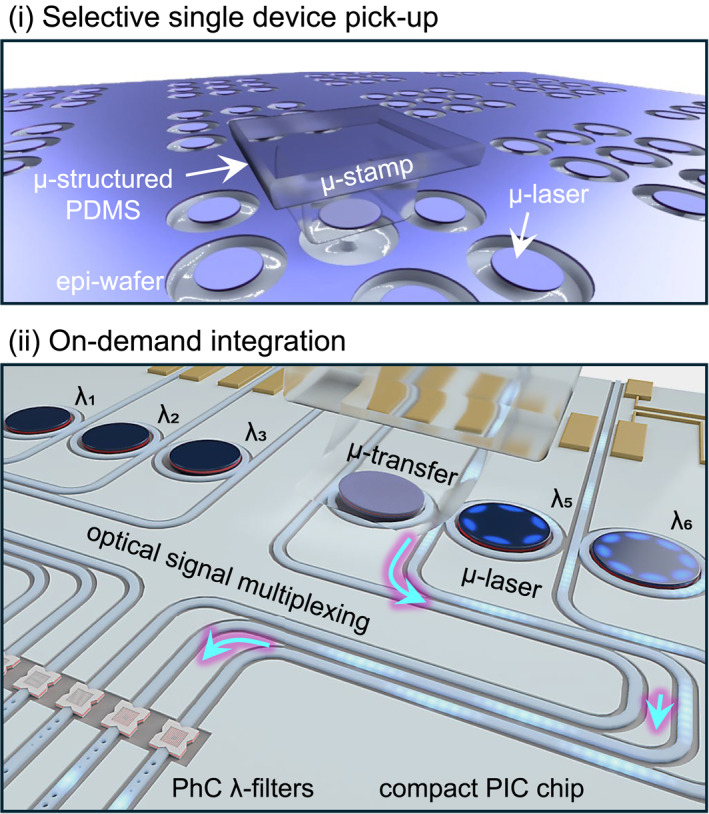

## 1 Introduction

The advancement of integrated photonic systems is increasingly driven by the demand for ultracompact, energy‐efficient, and scalable light sources that can operate seamlessly across heterogeneous material platforms [1, 2, 3, 4, 5, 6, 7, 8, 9, 10]. As information technologies extend beyond the limitations of electronic scaling, the integration of wavelength‐scale optical emitters with silicon photonics, compound semiconductors, and emerging quantum materials has become a critical challenge. This integration is essential for high‐bandwidth communication low‐power interconnects and quantum information processing [11, 12, 13, 14, 15, 16, 17, 18, 19, 20]. However, intrinsic material mismatches such as lattice constants, thermal properties, and the fabrication incompatibility, pose significant obstacles to direct epitaxial growth and wafer‐scale bonding approaches [21, 22, 23, 24, 25, 26, 27, 28, 29, 30, 31]. These limitations necessitate alternative strategies that can navigate rigid process constraints while maintaining nanoscale precision and scalability.

In this context, polydimethylsiloxane (PDMS)‐based micro‐transfer‐printing has emerged as a transformative methodology [32, 33, 34, 35, 36, 37, 38]. Transfer‐printing provides a versatile pathway to heterogeneously integrated photonic systems by enabling the deterministic release, pick‐up, and placement of pre‐fabricated light‐emitting structures onto arbitrary substrates. This innovative approach, distinct from conventional integration methods, enables the precise positioning of wavelength‐scale cavities nanobeams nanowires (NWs) and quantum emitters with sub‐micrometer accuracy, while maintaining their optical quality and functionality [39, 40, 41, 42, 43, 44] The technique not only optimizes material utilization but also separates the fabrication processes of gain media and passive photonic circuits, thereby providing unprecedented design flexibility.

This study consolidates recent advancements in transfer‐enabled integration of wavelength‐scale light sources, with a specific emphasis on PDMS‐assisted micro‐ and nanoscale devices. We explore the integration of microdisk and microring light sources, photonic crystal nanobeam lasers, NW emitters, and solid‐state quantum light sources, each presenting unique opportunities and challenges associated with transfer‐printing. Through a systematic analysis of these cases, we demonstrate how this technology balances throughput with deterministic precision, and how it is approaching a critical threshold for scalable integration of quantum emitters and single‐photon devices.

Beyond the immediate technical advancements, the implications of transfer‐enabled integration are far‐reaching, impacting areas, such as optical interconnects, for data centers, neuromorphic and AI hardware [45, 46, 47, 48], biosensing [49, 50], and defense applications [51, 52], where scalable and energy‐efficient light sources are essential. Notably, the inherent compatibility of PDMS‐based printing with CMOS processes [43] and its adaptability to three‐dimensional and programmable architectures [53, 54, 55, 56] indicate a clear pathway toward practical manufacturing. Consequently, this study highlights recent milestones and focuses on the significance of transfer‐enabled integration on the next generation of scalable photonic and quantum technologies.

## 2 Transfer‐Enabled Photonic Integration Technologies

**FIGURE 1:**
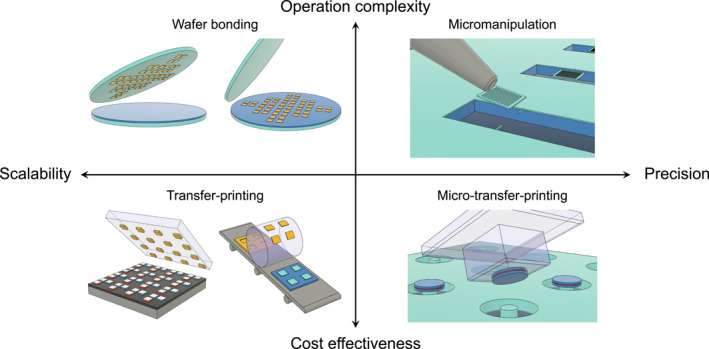
State‐of‐the‐art transfer‐enabled photonic integration technologies. Novel photonic integration technologies based on various transfer mechanisms are categorized by integration precision, scalability, operation complexity, and cost effectiveness: wafer‐ or die‐to‐wafer bonding (top, left), roll‐to‐roll stamping and large‐area transfer‐printing (bottom, left), micromanipulation‐based pick‐and‐place (top, right), and structured stamp‐based micro‐transfer‐printing (bottom, right).

For decades, numerous approaches have been proposed and experimentally validated for the development of high‐quality (high‐Q) micro‐ and nanoscale light sources [57, 58, 59], along with their efficient integration into compact photonic integrated circuits (PICs) [39, 40, 41, 42, 60, 61, 62, 63, 64, 65, 66, 67, 68, 69, 70, 71, 72, 73, 74, 75, 76, 77, 78, 79, 80, 81, 82, 83, 84, 85]. Various key technologies based on heterogeneous transfer mechanisms, each offering distinct advantages and practical benefits, have further fueled interest in transfer‐enabled photonic integration [6, 39, 40, 41, 42, 62, 63, 64, 65, 66, 67, 68, 69, 70, 71, 72, 73, 74, 75, 76, 77, 86, 87, 88, 89, 90, 91, 92, 93, 94, 95, 96, 97, 98]. Schematic illustrations of state‐of‐the‐art transfer‐enabled photonic integration technologies are shown in Figure 1. These technologies can be appropriately categorized based on their predominant strengths, such as scalability, precision, operational complexity, and cost effectiveness.

Among these methods, the wafer bonding technique, particularly when combined with post‐fabrication processes for III–V devices, is considered a widely adopted approach for the robust integration of III–V semiconductor light source arrays with conventional integration platforms (e.g., Si, Si_3_N_4_, and SiO_2_) at the wafer scale [1, 2, 3, 4, 5, 6, 22]. This technique typically utilizes the adhesive properties of a polymer layer (e.g., benzocyclobutene) or enhanced spontaneous adhesion generated at the interface between a surface‐treated semiconductor and an oxide layer [1, 2, 3, 4, 6, 23]. Numerous successful demonstrations have been reported, including the wafer bonding and microfabrication of InGaAsP and AlGaInP epitaxial III–V dies and chips, which contain rich optical gain structures, such as multi‐quantum‐wells (MQWs) [39, 40, 41, 62, 64, 65, 67, 68, 69, 70]. These have been transformed into arrays of high‐Q optical microresonators (e.g., microrings, microdisks, and Fabry–Pérot waveguides) and subsequently developed into electrically and/or optically driven low‐threshold micro‐ and nanolasers [78, 79, 80, 81, 82, 83, 84, 85]. Light is evanescently coupled to passive waveguides in a controllable manner by adjusting the thickness of polymer or oxide layers [99, 100]. This method presents significant advantages in scalability and mass production, highlighting its potential for direct industrial applications. However, several challenges persist regarding operational complexity and integration precision. The process often necessitates surface planarization with critical depth control of the receiving substrate [99], followed by a controlled oxidation process or low‐temperature surface treatments. Furthermore, to achieve strong and robust bonding, a series of dedicated post‐annealing processes must be conducted prior to the fabrication of III–V devices. In terms of integration precision, the bonding procedure initiates with the transfer of dies or chips onto the integration substrate, which requires an initial rough alignment [86]. The final precision and alignment are subsequently determined by the III–V device fabrication steps, including aligned lithography and etching. Consequently, for large wafer‐scale integration, the precision and alignment between individual optical components are inherently limited, often resulting in misalignments ranging from several hundred nanometers to a few micrometers. Furthermore, this method necessitates extensive integration sites, and methodologically renders pretesting of individual III–V devices impossible, resulting in significant waste of III–V materials and low overall device yields through the process. It also results in relatively low materials utilization efficiency at the device level, when large‐area III–V dies are bonded and subsequently patterned. These challenges impose critical limitations on the feasibility of on‐demand nanoscale light source applications in compact PICs.

Conversely, the pick‐and‐place integration method, which utilizes electronically controlled micromanipulators in conjunction with scanning electron microscope (SEM) imaging, provides substantial advantages in achieving high‐precision and nanoscale alignment [70, 88, 89, 95, 101, 102, 103]. This approach involves the fabrication of laser devices on an epitaxial growth wafer, followed by individual addressing, pick‐up, and precise alignment transfer using a micromanipulator. Consequently, the challenges associated with wafer‐bonding, such as post‐device‐fabrication complexity, integration precision, inefficient use of integration area, and material waste, can be significantly simplified and addressed methodologically. Notably, real‐time observation with high‐resolution SEM enables deep nanoscale alignment, thereby facilitating advanced on‐demand integration that is sensitive to the structural arrangements and critical optical couplings in compact PICs. This includes direct and/or evanescent coupling between low‐dimensional semiconductor light‐emitting nanomaterials, nanocavity lasers, and various types of Si or Si_3_N_4_ optical waveguides [88, 95, 104]. However, this approach presents notable drawbacks, including low cost‐effectiveness and high operational complexity. The SEM‐based micromanipulator system is a substantial and costly apparatus, with limited economic viability. Additionally, the series of delicate processes conducted in a vacuum environment with high electron energies, including selective‐area cutting, sectioning, corner‐picking, repeated orientation and alignment, and positional adjustments, require highly skilled personnel and significantly prolonged operation times, rendering the technique less favorable for broad application. Furthermore, rigid micromanipulators can inflict considerable physical damage to materials and structures during manipulation, resulting in undesirable degradation of optical properties and overall device performance.

Meanwhile, the transfer‐printing technique has garnered considerable attention as a promising integration method [39, 40, 41, 42, 60, 62, 63, 64, 65, 66, 67, 68, 69, 70, 71, 72, 73, 74, 75, 76, 77, 105, 106, 107, 108, 109, 110, 111]. This approach employs an optically transparent, mechanically flexible, and adhesive polymeric stamp, facilitating pick‐and‐place operations with minimal physical damage and degradation on III–V devices under a conventional optical microscope. Notably, the entire transfer setup features a compact design, requiring minimal space. Integration is achieved through a straightforward two‐step process, conducted under real‐time observation, enhancing both the economic viability and user‐friendliness of the technique. PDMS is a predominant material for the stamp owing to its ease of fabrication, controllable adhesiveness, and exceptional flexibility. A key feature of this method is a kinetic control mechanism, which serves as the working principle that leverages changes in adhesiveness based on the speed of peel‐off and place‐on processes [105, 106, 107, 108]. This capability allows for a wide range of integrations, from transfer‐printing patterned arrays of different colors of light‐emitting materials for highly efficient micro‐LEDs [36, 38, 63, 112, 113, 114, 115, 116, 117, 118, 119, 120, 121], to wafer‐scale roll‐to‐roll transfer for flexible [122] and transparent display applications [123, 124, 125]. The scalability of this technique ensures mass production capabilities, thereby enhancing cost‐effectiveness and demonstrating significant potential for industrial applications. However, despite these advantages and proven success, challenges persist in the integration of compact PICs, where precise on‐demand integration of individual micro‐ and nanoscale light sources is essential. In this context, micro‐transfer‐printing has emerged as a state‐of‐the‐art integration method in recent years [41, 68, 69, 70, 71]. Leveraging the numerous advantages of transfer‐printing, this technique particularly emphasizes key features, such as individually and selectively addressable transfers, along with highly precise on‐demand integration facilitated by nanoscale alignment.

**FIGURE 2:**
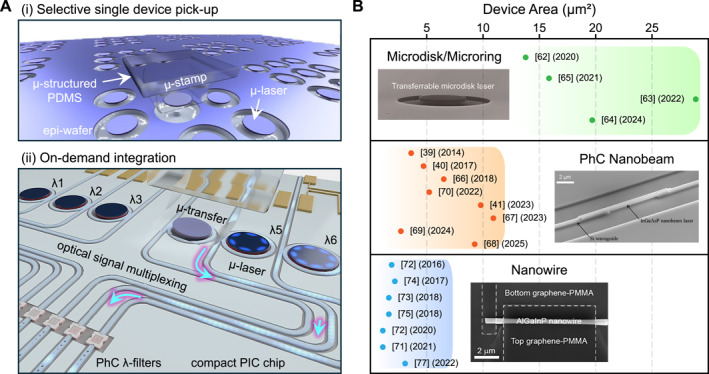
Key features of micro‐transfer‐printing and demonstrations of wavelength‐scale light sources. (A) Schematics of characteristic sequence of micro‐transfer‐printing: (i) individually and selectively addressable pick‐up of a microdisk laser on an epitaxial growth wafer, (ii) on‐demand integration at predetermined locations on the compact PIC chip. (B) Overview of transfer‐printed wavelength‐scale light sources, categorized into three representative classes: microdisks/microrings, photonic crystal nanobeams, and semiconductor nanowires, plotted as a function of their device footprints.

A schematic representation of several key features of micro‐transfer‐printing‐enabled integration is shown in Figure 2A. The PDMS stamp is appropriately microstructured to enable the individual and selective pick‐up of targeted devices, ensuring that other devices remain undamaged and uncollected. Here, III–V semiconductor laser devices, akin to those utilized in pick‐and‐place methods, are fabricated separately on a growth wafer. Notably, the mechanical properties of the PDMS stamp are carefully tailored during fabrication to optimally facilitate transfer‐integration of the target device. In certain instances, mild surface modification through oxidation processes is employed to fine‐tune the levels of flexibility and adhesiveness [37, 126, 127, 128, 129, 130, 131, 132]. The device is then brought onto a compact PIC chip, where time‐controlled contact and peel‐off steps are subsequently performed to achieve precise on‐demand integration at predesignated locations. This integration occurs within a confined area, ensuring that no other portions or components of the PIC are physically and optically perturbed during the process. In addition to demonstrating the fundamental working principle of micro‐transfer‐printing in Figure 2A,B provides a comparative overview of transfer‐printed wavelength‐scale light sources, categorized into three major classes—microdisks/microrings, PhC nanobeams, and semiconductor NWs—plotted against their respective transferred device footprints. Each category features unique cavity geometries and characteristic dimensions, ranging from a few micrometers in diameter for microdisks and microrings, to sub‐micrometer widths and approximately 10 μm lengths in PhC nanobeams, and diameters below 500 nm in NWs. These dimensions underscore the exceptional precision required for deterministic pick‐up and placement of fragile structures. By leveraging the kinetic adhesion control of PDMS stamps, these devices can be reliably detached from their native substrates and reassembled onto silicon, silica, polymeric, or metallic platforms with minimal optical degradation. This capability facilitates the scalable integration of ultracompact and functional light sources that surpass the limitations of conventional bonding or epitaxial growth techniques.

A comprehensive overview of these demonstrations is outlined in Table 1, which summarizes the key advances across all three categories of wavelength‐scale emitters realized by transfer printing, with particular emphasis on their integration strategies and coupling configurations within diverse photonic platforms. For microdisks and microrings, PDMS‐assisted transfer printing preserves high‐Q whispering‐gallery modes (WGMs) while enabling deterministic placement on planar photonic substrates. These approaches support both optically and electrically driven operation, including silicon‐post‐supported microdisks, electrically addressable devices employing transparent graphene contacts, and gain‐patch‐assisted hybrid silicon microlasers, collectively demonstrating the versatility of transfer printing for compact WGM‐based light sources in integrated photonic systems. In the case of PhC nanobeam lasers, Table 1 highlights a systematic progression of light‐extraction and coupling strategies enabled by transfer printing. These include vertically stacked evanescent coupling to silicon‐on‐insulator (SOI) waveguides, docking‐assisted and side‐coupled integration onto Si_3_N_4_ circuits, and engineered unidirectional on‐waveguide configurations, as well as three‐dimensional coupling schemes involving optical fibers. Such approaches allow efficient interfacing with guided or free‐space photonic modes while relaxing alignment constraints and preserving strong optical confinement, leading to record‐low lasing thresholds and room‐temperature continuous‐wave operation. More recently, gain‐patch‐assisted nanobeam architectures further extend this concept by transferring only minimal III–V gain regions onto passive cavities, underscoring the flexibility of transfer printing for heterogeneous photonic integration beyond conventional PIC layouts. For NWs, transfer printing plays a distinct yet complementary role by addressing the inherent positional and performance variability of bottom‐up grown ensembles. As summarized in Table 1, deterministic selection, orientation, and ordered placement of individual nanowires enable dense serial integration, programmable emission control, vertical emission engineering, and controlled coupling to planar waveguides, polymeric photonic structures, or fiber‐based platforms. Beyond optically pumped operation, these strategies also support electrically injected nanowire sources integrated with photonic guiding elements, highlighting a pathway toward practical on‐chip and hybrid photonic light sources based on transfer‐printed NW devices. Overall, the results compiled in Table 1 emphasize the breadth of photonic integration platforms and coupling modalities achievable with PDMS micro‐transfer printing, spanning CMOS‐compatible silicon photonics as well as unconventional polymeric, free‐space, and fiber‐based systems. At the same time, they demonstrate the scalability and precision of this technique in handling devices with footprints down to a few hundred nanometers, thereby providing a unified perspective on how transfer‐printed wavelength‐scale emitters have evolved from isolated devices toward broadly integrated photonic architectures. Moreover, this integration technology has also proven its applicability to diverse functional substrates including glass/ITO/HTL, flexible polymer films, as well as unconventional platforms such as woods, leaves, and papers [36, 37, 133].

**TABLE 1:** Summary of transfer‐printed wavelength‐scale light sources.

Ref.	Device type	Material	Footprint	Integration strategy	Operation
[62]	Microdisk (laser)	InGaAsP MQW	D 4.2 μm × T 0.26 μm	Post‐supported microdisk integration on SOI (no direct WG coupling)	Optical/pulsed (RT)
[63]	Microdisk (LED)	AlGaInP MQW	D 6 μm × T 0.2 μm	Electrically addressable microdisk integration with graphene contacts	Electrical/CW (RT)
[64]	Microdisk (laser)	InGaAsP MQW	D 5 μm × T 0.22 μm	Meta‐micromirror‐assisted free‐space out‐coupling of transfer‐printed microdisk lasers	Optical/pulsed (RT)
[65]	Microring (laser)	InGaAsP MQW	D 2.5 μm × W 0.5 μm × T 0.18	Gain‐patch‐assisted hybrid silicon microlaser formed by transfer‐printed III–V gain	Optical/pulsed (RT)
[39]	PhC nanobeam (laser)	InGaAsP MQW	W 0.6 μm × L 6.6 μm× T 0.28 μm	Substrate‐level integration of a free‐standing nanobeam on SiO_2_/Si (PIC‐compatible baseline)	Optical/CW (RT)
[40]	PhC nanobeam (laser)	InGaAsP MQW	W 0.58 μm × L 8.0 μm × T 0.28	Vertically stacked directional evanescent coupling to an SOI WG	Optical/pulsed (RT)
[66]	PhC nanobeam (laser)	InAs/GaAs QDs	W 0.472 μm × L ∼13.9 μm	Evanescent coupling to a CMOS silicon WG enabling multiple nanolasers on a single WG	/Pulsed (8K)
[41]	PhC nanobeam (laser)	InGaAsP MQW	W 0.6 μm × L ∼16 μm × T 0.24 μm	Docking‐assisted side coupling onto a SiN_x_ WG with WG‐assisted alignment	Optical/pulsed (RT)
[67]	PhC nanobeam (laser)	InGaAsP MQW	W 0.6 μm × L∼18 μm × T 0.24 μm	On‐WG integration with engineered unidirectional coupling into a SiN_x_ WG port	Optical/pulsed (RT)
[68]	PhC nanobeam (laser)	InGaAsP MQW	W 0.6 μm × L ∼16 μm × T 0.24 μm	Side‐coupled hybrid integration to a silicon WG using asymmetric cavity–WG coupling	Optical/pulsed (RT)
[69]	PhC nanobeam (laser)	InGaAsP MQW	W 0.66 μm × L 4.6 μm × T 0.22 μm	Gain‐patch‐assisted laser‐on‐WG architecture on a silicon PhC cavity	Optical/CW (RT)
[70]	PhC nanobeam array (laser)	InGaAsP MQW	W 0.58 μm × L 9 μm × T 0.2 μm	Three‐dimensional nanobeam array transfer‐printed on an optical microfiber for programmable WG coupling	Optical/pulsed (RT) (programmable)
[71]	Nanowire (laser)	AlGaAs, diamond, GaN; InP, GaAs/AlGaAs	D 0.26–0.45 μm × L 4–10 μm	Dense serial transfer printing of heterogeneous μ‐scale devices on shared WGs	Optical/pulsed (RT)
[72]	Nanowire array (laser)	GaAs–AlGaAs core–shell NWs	D 0.45 μm × L 4 μm	Threshold‐based selection and deterministic pick‐and‐place assembly of nanowire lasers	Optical/pulsed (RT)
[42]	Nanowire (single, bundles, arrays) (laser)	InP NW	D 0.84–0.89 μm; L few μm	Evanescently coupled nanowire laser transfer‐printed onto planar WGs	Optical/pulsed (RT)
[73]	Nanowire (laser)	InP NW	D 0.43−0.92 μm; L few μm	Deterministic transfer printing of nanowire lasers for PIC‐ready integration	Optical/pulsed (RT)
[74]	Nanowire (laser)	InP NW (hexagonal)	D 0.435 μm × L 6 μm	SU‐8 polymer WGs on SiO_2_/flexible glass	Optical/pulsed (RT)
[75]	Nanowire (laser)	InP NW + Al CE nanoantenna	D 0.29 μm × L 0.9 μm	Nanoantenna‐assisted vertical emission from transfer‐printed nanowire lasers	optical/pulsed (7K)
[76]	Microwire (PT‐symmetry)	GaN hexagonal microwire	D ≈ 1 μm, L Few μm (tapered)	Direct polaritonic mode coupling in a single microcavity via loss‐engineered integration	Optical/pulsed (RT)
[77]	Nanowire (LED)	AlGaInP NW + graphene p/n contacts (MQWs)	D 0.3 μm × 9 μm	Electrically pumped nanowire source integrated with planar WGs	Electrical/CW (RT)

## 3 Integration of Wavelength‐Scale Light Sources

### 3.1 Microdisk and Microring Light Sources

**FIGURE 3:**
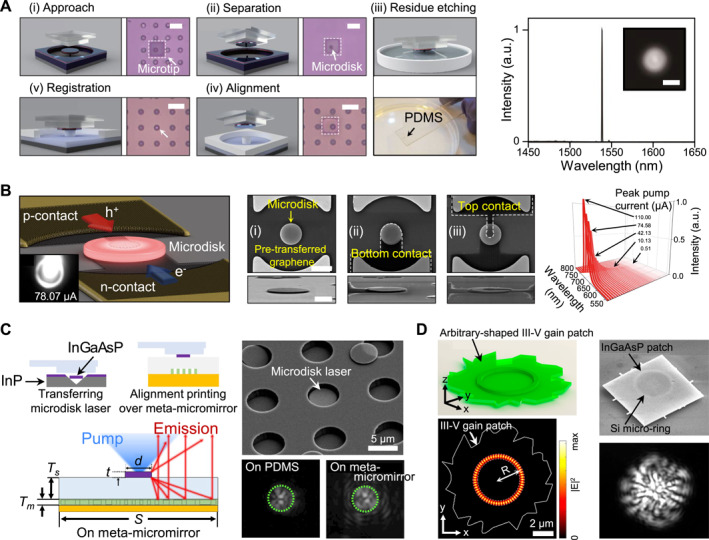
Integration of microdisk and microring light sources through PDMS‐based transfer‐printing. (A) On‐chip transferrable microdisk laser integrated onto a silicon post utilizing PDMS microtransfer printing, demonstrating low‐threshold lasing with preserved whispering‐gallery modes. (B) Electrically driven microdisk light source achieved by combining vertical *p*‐*i*‐*n* microdisks with transparent graphene electrodes, highlighting the feasibility of current‐injected transferrable devices. (C) Microdisk laser integrated with a meta‐micromirror through transfer printing, enabling directional emission control and enhanced collection efficiency. (D) Hybrid microring laser formed by transfer‐printing arbitrary‐shaped InGaAsP gain patches onto pre‐fabricated silicon microring resonators, demonstrating design‐flexible heterogeneous laser integration, reproduced with permission from Refs. [62, 65], American Chemical Society (A), (D), Ref. [63], American Institute of Physics (B), and Ref. [64], De Gruyter (C).

Among the various light sources investigated through transfer‐enabled integration, microdisk and microring light sources have emerged as significant case studies. Their circular resonator geometries facilitate strong optical confinement through WGMs, whereas their compact footprints render them particularly suitable for heterogeneous integration with silicon photonics. The representative demonstrations of the impact of PDMS‐assisted transfer‐printing of the deterministic assembly and functional enhancement of such devices are shown in Figure 3.

The pioneering demonstration that leverages PDMS micro‐transfer‐printing to release and reassemble InGaAsP microdisk lasers onto silicon posts fabricated on SOI substrates is shown in Figure 3A [62]. During the transfer process, the elastomeric and transparent PDMS stamp enabled the selective detachment of pre‐fabricated III–V microdisks from their growth substrate, followed by precise placement with minimal mechanical stress. Notably, this approach preserved the high‐Q factor WGMs essential for lasing, even after the transfer to an air‐semiconductor interface supported by a Si post. Optical characterization revealed lasing action with thresholds as low as approximately 97 μW, validating the potential of transferrable microdisks as efficient and compact on‐chip light sources. Building on this proof of concept, researchers have advanced transfer‐printing methods to realize electrically driven microdisk light sources, as shown in Figure 3B [63]. In this approach, vertically doped AlGaInP microdisks containing multiple quantum wells were fabricated and subsequently transferred onto Si_3_N_4_ substrates. A significant challenge in scaling these devices is the integration of reliable, non‐obstructive electrodes. This issue was effectively addressed by introducing mechanically flexible, optically transparent multilayer graphene (MLG) contacts on both the top and bottom surfaces of the microdisks. This configuration facilitates efficient current injection while preserving optical confinement. Electroluminescence experiments demonstrated diode‐like I–V characteristics, polarization‐resolved emission spectra, and robust operational stability, thereby validating that micro‐transfer‐printing can support both optically pumped and current‐driven III–V light sources on photonic integration platforms.

Another integration strategy, as shown in Figure 3C, involves the hybrid coupling of microdisks with a metasurface, specifically implemented as a meta‐micromirror, which enables versatile beam shaping and enhances emission characteristics [64]. In this configuration, a microdisk laser was printed above a reflective dielectric metasurface patterned on a gold mirror, separated by a polymer spacer (SU‐8). The metasurface was designed to redirect the otherwise divergent emission from the microdisk into a near‐vertical trajectory. Consequently, the collection efficiency within a numerical aperture (NA) of 0.65 is enhanced by a factor of 2.68 compared with that of bare disks.

Recently, a novel paradigm of integration that decouples resonators from the gain medium has emerged, allowing for unprecedented design flexibility. As shown in Figure 3D, ultrathin (approximately 180 nm) InGaAsP gain patches containing multiple quantum wells were transfer‐printed directly onto pre‐fabricated silicon microring resonators [65]. Notably, lasing was achieved regardless of the precise size, shape, or lateral placement of the gain patch, attributed to the strong optical confinement provided by the silicon cavity modes. This finding demonstrates that microrings fabricated entirely through CMOS‐compatible processes can be transformed into lasers simply by affixing appropriately engineered III–V gain patches. This gain‐patch printing technique not only minimizes material consumption but also provides a sophisticated approach to post‐fabrication customization of photonic circuits, enhancing design flexibility and easing alignment constraints.

### 3.2 Photonic Crystal Nanobeam Light Sources

**FIGURE 4:**
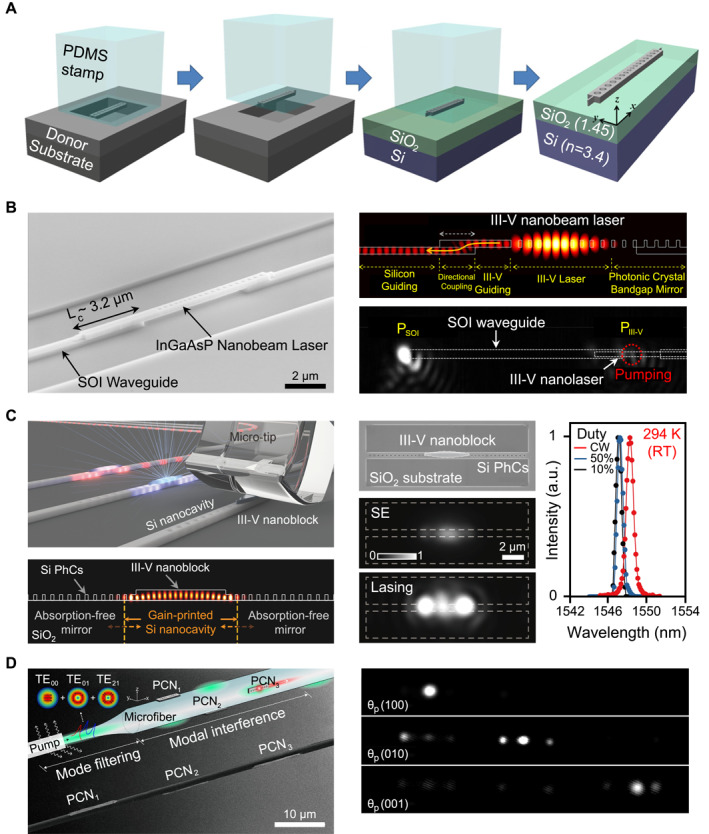
Integration of photonic crystal nanobeam light sources through PDMS‐based transfer‐printing. (A) Transfer‐printed InGaAsP photonic crystal nanobeam laser fabricated as a tethered free‐standing cavity and transferred onto Si, demonstrating low‐threshold continuous‐wave lasing at room temperature. (B) High‐efficiency hybrid integration of a nanobeam laser onto a silicon‐on‐insulator waveguide, achieving coupling efficiency up to 83% through optimized asymmetric cavity design. (C) Minimal‐gain–printed Si nanolaser formed by transfer‐printing a nanoscale InGaAsP gain block onto a Si photonic crystal cavity, enabling room‐temperature continuous‐wave operation with enhanced thermal stability. (D) Three‐dimensional programmable nanobeam laser arrays transfer‐printed on an optical microfiber, where selective activation of individual lasers is achieved through modal interference of the pump beam. Reproduced with permission from Refs. [39, 70], Optica Publishing Group (A), (D), Ref. [40] American Chemical Society (B), Ref. [69] American Association for the Advancement of Science (C).

In addition to the advancements in microdisk and microring light sources, significant efforts have been directed toward PhC nanobeam lasers. These devices feature even smaller mode volumes and lower thresholds, rendering them highly suitable for compact photonic integration. Recent advancements in transfer‐printing‐enabled integration of these nanobeam devices are shown in Figure 4. The architectures and functionalities are shown in the figure.

The transfer‐printing process of a typical PhC nanobeam laser, where InGaAsP PhC nanobeam cavities are fabricated as free‐standing membranes by introducing tether structures at both ends of the beam and releasing them through selective wet etching, is shown in Figure 4A [39]. Utilizing a PDMS micro‐stamp, the fragile beams were detached from their original substrates and transferred onto target chips without adhesives. The printed nanobeam, characterized by a mode volume of approximately 10.5 ${\left(\lambda /n\right)}^{3}$, demonstrated continuous‐wave lasing near 1550 nm at room temperature, achieving a remarkably low threshold of approximately 9.0 μW.

To further enhance device functionality, a strategy for efficient coupling was developed, as shown in Figure 4B [40]. In this approach, a transfer‐printed III–V nanobeam laser was meticulously aligned onto an SOI waveguide. By employing an asymmetric one‐dimensional PhC cavity design, the nanobeam emitted light unidirectionally into an adjoining InGaAsP waveguide, which vertically overlapped with the SOI waveguide to form a compact directional coupler. This configuration achieved an experimental coupling efficiency of up to 83%, representing a highly efficient demonstration of direct III–V/Si nanolaser integration. Notably, unidirectional coupling based on the asymmetric design principle has also been demonstrated for Si_3_N_4_ waveguides using transfer‐printed nanobeam lasers [67].

A novel integration paradigm was subsequently introduced, wherein the cavity was constructed entirely in silicon, with only a nanoscale gain block printed at its center, as shown in Figure 4C [69]. In this approach, a tapered InGaAsP nanoblock containing multiple quantum wells was transfer‐printed onto a one‐dimensional Si PhC nanobeam cavity. This minimal‐gain design effectively confined carriers, suppressed parasitic absorption, and leveraged the superior thermal conductivity of silicon. Consequently, lasing was achieved under room‐temperature continuous‐wave excitation, successfully addressing longstanding challenges related to thermal instability and excessive threshold power. This strategy exemplifies a scalable, Si‐native architecture that minimizes the use of active III–V materials in the lasing mode volume. Notably, a conceptually related approach has also been reported using subwavelength ZnO nanowires embedded in linear grooves and surrounded by SiN two‐dimensional photonic crystal waveguides, where nanowire‐induced nanocavities employing a minimal gain volume exhibited low‐threshold lasing up to elevated temperatures [134].

Beyond planar integration, transfer‐printing has also enabled the development of unconventional three‐dimensional laser architectures. As shown in Figure 4D, arrays of PhC nanobeam lasers are deterministically printed onto the sidewalls of a tapered optical microfiber [70]. By utilizing modal interference of the pump beam guided through the fiber, individual nanolasers within the array could be selectively and programmably activated. Adjusting the polarization, phase, or pulse width of the pump, different subsets of lasers could be engaged, allowing for complete optical control of the array. This fiber‐based platform highlights the potential for compact, programmable multiwavelength sources applicable in areas such as wavelength‐division multiplexing photonic circuits, biosensing, and quantum networks.

### 3.3 Nanowire Light Sources

Semiconductor NWs represent a compelling class of nanomaterials for wavelength‐scale light sources [42, 71, 72, 73, 74, 75, 76, 77, 135, 136, 137, 138, 139, 140, 141, 142, 143, 144, 145]. Their material versatility allows for the synthesis of diverse III–V and II–VI compound NWs (e.g., InP, GaAs, GaN, ZnO, CdS, and CsPbBr_3_) that emit across the visible and near‐infrared frequencies [90, 135, 146, 147, 148, 149, 150, 151, 152, 153]. Synthetic controllability allows for the creation of periodic optical gain structures, such as MQWs, as well as rationally engineered core/shell and/or axial *p*‐type/intrinsic/*n*‐type heterostructures [139, 140, 154, 155, 156, 157, 158, 159], thereby facilitating device fabrication capabilities [155, 160, 161, 162, 163]. The compact dimensions of NWs, with diameters ranging from approximately 0.1–1 μm and lengths between 1 and 20 μm, combined with their high material gain, render them particularly advantageous for nanoscale integration and efficient light generation [138, 164]. This supports robust on‐chip coupling to essential components of nanophotonic circuits. For example, various II–VI and III–V NWs have been demonstrated as nanoscale lasers [42, 73, 150] and micro‐LEDs [77], often achieving continuous‐wave coherent emission at low thresholds [165, 166] and demonstrating stable operation at room temperature [76, 152]. These characteristics position them as practical on‐chip emitters for integrated nanophotonic systems [74, 151, 167]. However, scalable integration poses a significant challenge within the NW research community owing to the inherent randomness in the position and orientation of as‐grown NWs. Addressing this challenge typically necessitates the pre‐screening of optically active NWs and their deterministic alignment to predefined coupling sites, such as waveguide top facets or narrow gaps for evanescent coupling, while maintaining intrinsic optical properties and device performance, and avoiding disturbances to previously integrated devices. PDMS‐based nano‐/micro‐transfer‐printing effectively addresses this requirement by enabling the selective pick‐and‐place process for prefabricated pre‐screened NWs with sub‐micrometer alignment accuracy and high yield [71, 72]. In the following section, we outline several strategies that leverage PDMS micro‐stamps for the precise transfer of individual NWs onto diverse receiving substrates. We also highlight representative device integrations where transfer‐printing significantly enhances the performance of stand‐alone and waveguide‐coupled nanoscale lasers and emitters, nanostructure‐coupled hybrid devices, and graphene‐contact micro‐LEDs.

**FIGURE 5:**
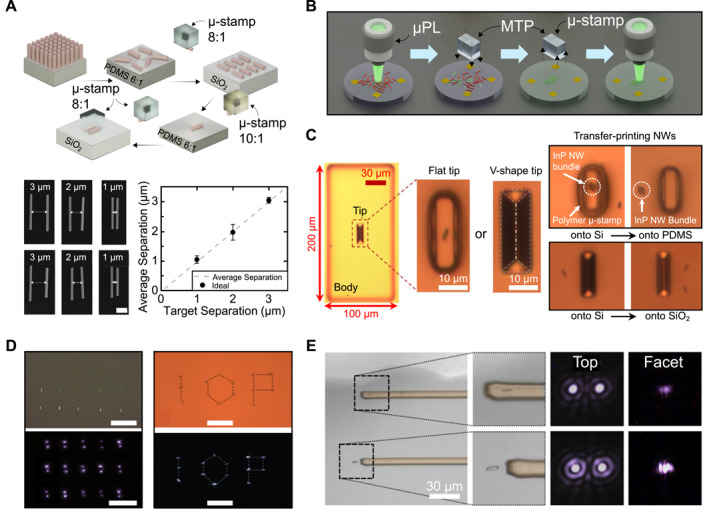
Transfer‐printed nanowire lasers utilizing PDMS μ‐stamps. (A) As the integration process of NW devices, NWs are transferred from the growth substrate onto a target surface, a configuration achieved with laterally aligned pairs. (B) Schematic of the NW laser transfer and integration process utilizing PDMS μ‐stamps, from characterization to selective printing and recharacterization. (transfer process: 1: characterized and sorted NW lasers on a quartz disk, 2: selectively picked with a PDMS μ‐stamp, 3: printed into arrays, 4: recharacterized). (C) Design of PDMS flat/elongated pyramidal ‐tip μ‐stamp tips. Top: flat‐tip μ‐stamp aligned with an InP NW bundle underlying Si (left), after printing the NW bundle on PDMS (right). Bottom: elongated pyramidal tip μ‐stamp adjacent to an InP NW lying down on Si (left), and printed NWs on silica (right). (D) Two‐dimensional arrays of InP NW lasers fabricated by nano‐transfer‐printing on a PDMS substrate with aligned rows, shown as a collage of lasing NW images (left), and forming the IOP pattern (Institute of Photonics). (E) SU‐8 waveguides on flexible glass integrated with NW lasers in the lateral (top) and facet (bottom) coupling configurations. The insets represent magnified areas and lasing emission (VS‐collected) from the NWs, along with corresponding facet images. Reproduced with permission from Ref. [71], Optica Publishing Group (A), from Refs. [42, 72, 74], American Chemical Society (B), (C), (E), from Ref. [73] The Institution of Engineering and Technology, (D).

To address aforementioned issues, PDMS‐based micro‐transfer‐printing using micro‐stamps has been developed. As shown in Figure 5A these microstructured stamps effectively retrieve single NWs from their growth substrates and place them in a well‐ordered array with micrometer‐scale pitch size (approximately 1–3 μm) while ensuring precise alignment with target sites [71]. Here, the adhesion of the stamp was meticulously optimized by adjusting the mixing ratio of the PDMS base to crosslinker and controlling the peel rate, with the following practical adhesion hierarchy: 10:1 PDMS ≥ SiO_2_ > 8:1 PDMS > 6:1 PDMS. This deterministic workflow transformed randomly oriented nanostructures into ordered arrays that are aligned to pre‐patterned photonic structures or user‐defined layouts, facilitating integration with on‐chip PICs. The process was further supported by a closed pre‐ and post‐transfer characterization loop (Figure 5B) [72]. Initially, NWs were pre‐screened using micro‐photoluminescence (μ‐PL) on auxiliary platforms to identify optically active, lasing‐capable devices; only the selected NWs were subsequently transfer‐printed. Following micro‐transfer‐printing, re‐characterization validated the laser threshold, peak wavelength, and polarization stability, thereby enhancing reproducibility in dense photonic assemblies. Furthermore, by finely tuning and controlling the adhesion and geometric shape of micro‐stamps, transfer‐printing becomes widely applicable across a range of substrates. For example, as shown in Figure 5C, both flat and elongated‐pyramidal (V‐shaped) tips successfully accommodate diverse surfaces of receiving substrates with varying surface energies (e.g., PDMS, Si, and SiO_2_) and facilitated access to narrow gaps near coupling structures [42]. These adhesion tunability and shape controllability enabled precise placement onto Si, SiO_2_, polymer films, and metallic pads (e.g., Au), supporting the intricate fabrication of both ordered arrays and custom patterns, such as letters (e.g., “IOP”), as shown in Figure 5D [73]. Representative InP NW lasers, selected by μ‐PL and aligned through micro‐stamp transfer, retained their lasing characteristics after transfer‐printing. When positioned at waveguide facets (end‐fire coupling) or transfer‐printed on top of SU‐8 polymer waveguides on flexible glass, light was efficiently coupled into the guides while maintaining stable emission under repeated measurements (Figure 5E) [74].

**FIGURE 6:**
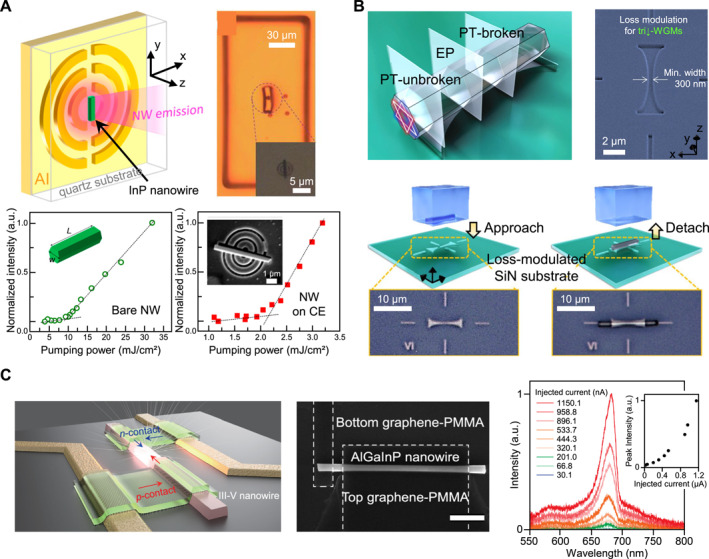
Advanced integration of nanowire lasers through precision transfer‐printing. (A) Vertical‐emitting InP nanowire‐cat's eye (NW‐CE) laser integrated by nano transfer‐printing. Compared with a baer nanowire, the CE structure provides vertical directionality, enhanced far‐field emission, and reduced lasing threshold. (B) GaN hexagonal microwire integrated on a loss‐modulated SiN substrate utilizing accurate PDMS μ‐stamp transfer. This platform enables precise optical integration and investigates polaritonic PT‐symmetry and other non‐Hermitian phenomena. (C) Electrically pumped III–V nanowire with top‐bottom all‐graphene contacts fabricated through PDMS transfer‐printing. This all‐graphene‐contact design demonstrates a transferrable nanowire laser with unique electrical injection properties. Reproduced with permission from Refs. [75, 77], American Chemical Society (A), (C), Ref. [76], Nature Publishing Group (B).

With deterministic micro‐stamp‐assisted placement established, the subsequent phase involves the integration of NWs into photonic devices. Recent studies have demonstrated that precision transfer‐printing facilitates the development of advanced and complex device architectures, demonstrating enhanced or unique optical properties when NWs are accurately positioned and registered on pre‐patterned photonic nanostructures with high accuracy. For example, as shown in Figure 6A, a single InP NW was precisely integrated at the center of a cat's‐eye (CE) nanoantenna, also referred to as a split bull's‐eye [168], utilizing a micro transfer‐printing technique. This method involved the monolithic definition of an array of CE patterns on a thin SiO_2_ layer deposited on quartz substrate through lithography and etching processes. Single NWs, pre‐selected for their lasing capabilities through a brief μ‐PL screening, were then deterministically positioned at the center of each CE pattern. The device fabrication was finalized with the deposition of a thin Al layer. Notably, the presence of several pairs of concentric semi‐circular plasmonic cavities or gratings enhanced the effective mirror reflectivity and cavity Q‐factor. This configuration facilitated strong guided‐mode focusing through surface‐plasmon‐assisted lens effects and enhanced spontaneous emission coupling, known as the Purcell effect. These enhancements resulted in reduced round‐trip loss, increased mode‐gain overlaps, and enabled highly vertical, collimated far‐field emission with enhanced polarization purity and a lower lasing threshold compared with that of a bare NW [75].

Another example of intriguing optical behaviors is shown in Figure 6B. The schematic and SEM images indicate a slightly tapered hexagonal GaN microwire, which is deterministically micro‐transfer‐printed onto a loss‐modulated Si_3_N_4_ substrate to form a room‐temperature polaritonic platform [76]. In this configuration, a specifically designed double hourglass‐shaped trench was incorporated into the high‐index substrate, and the axis of the tapered microwire carefully aligned with the trench axis during integration. This architecture yields a continuously varying air‐microwire interface along the wire axis, which inherently introduces a spatially modulated optical loss for the modes supported by the cross‐section of the microwire. Position‐ and power‐resolved μ‐PL measurements have effectively demonstrated polariton condensation with emission wavelengths near 360–370 nm. Selective excitation utilizing a high‐NA objective has provided a detailed mapping of the transition from the edge, characterized by low loss from large air–wire interface, to the center, characterized by high loss from a small air–wire interface, along the printed wire. As the engineered loss increases, the system transitions from a parity‐time (PT)‐unbroken regime to a PT‐broken phase through an exceptional point (EP). Direct coupling between degenerate triangular polariton modes (upper and lower) emerges, and the EP behavior appears at modest pump powers. Consequently, the engineered structures highlighted the potential for on‐chip EP functionality, which can enhance frequency‐shift‐sensing schemes and leverage a steep dispersive response near EPs.

Next, achieving electrical operation represents a critical milestone for the practical application of nanoscale light sources in a compact PIC. Among various strategies, the integration of micro transfer‐printing with an all‐graphene‐contact scheme presents a promising approach for on‐chip implementation. As shown in Figure 6C, a top–down‐fabricated AlGaInP *p*‐type/intrinsic/*n*‐type (*p*‐*i*‐*n*) NW with MQWs designed for emission at approximately 680 nm was selectively picked up using a PDMS micro‐stamp and precisely positioned at a designated site on a Si_3_N_4_ platform. Subsequently, a mechanically flexible, optically transparent, and patterned MLG was introduced to both the top and bottom surfaces of the NW, serving as electrical contacts [77]. At room temperature, pulsed currents were injected into the NW device, and electroluminescence spectra were recorded. The device demonstrated a distinct diode‐like characteristic with a turn‐on voltage of approximately 8.4 V, validating that the transfer‐printing of an electrically doped NW onto a graphene contact facilitated the formation of a robust current path to operate the NW LED. Additionally, a single NW LED was micro‐transfer‐printed on top of a Si_3_N_4_ strip waveguide, showcasing the potential for individually addressable integration of a nanoscale light source, stable current injection, controlled light generation, and effective coupling of emitted light into photonic waveguides.

In summary, the results shown in Figures 5 and 6 highlight that PDMS‐based transfer‐printing converts randomly distributed NWs into deterministically positioned building blocks for photonic integration. By employing a closed pre‐ and post‐transfer characterization loop alongside adhesion tuning and micro‐stamp tips geometry control, individual NWs are selected, aligned, and registered to predefined coupling sites. This process enabled the creation of ordered arrays, user‐defined layouts, and efficient waveguide coupling (Figure 5A–E). With this placement precision as a foundation, advanced device architectures become accessible, including CE nanoantennas that collimate and reduce thresholds in vertical emitters, loss‐engineered Si_3_N_4_ platforms that reveal PT‐symmetry breaking in GaN microwires, and electrically driven III–V NWs realized with all‐graphene contacts on Si_3_N_4_ (Figure 6A–C). Collectively, these findings position NWs as compact dimensions with high material gain and deterministic placement, paving the way for transferable lasers and scalable on‐chip light sources for optical interconnects and emerging quantum‐photonic circuits in the realm of non‐Hermitian photonics. The following section extends to quantum light sources, outlining integration strategies and representative case studies, along with a discussion of practical implementation strategies.

## 4 Integration of Quantum Light Sources

### 4.1 III–V Quantum Dot Light Sources

For scalable quantum‐photonic integrated circuits (Q‐PICs), the seamless integration of deterministic and efficient single‐photon sources (SPSs) with passive photonic circuit components or platforms is essential. While nonlinear processes in PICs and solid‐state color centers provide viable alternatives [169, 170, 171], semiconductor quantum dots (QDs) are particularly notable for their capability to deliver bright, pure, and indistinguishable single photons [172, 173, 174]. A significant challenge remains in hybridizing III–V QDs with low‐index or CMOS‐compatible photonic circuits without compromising optical performance [43, 175, 176, 177]. Conventional methods, such as wafer bonding or epitaxial growth methods, often impose stringent material constraints, limited alignment tolerance, and low yields. Conversely, PDMS‐based micro transfer‐printing has recently emerged as a versatile technique for integrating pre‐fabricated QD SPSs onto various photonic substrates with sub‐micron precision. This approach decouples the fabrication of emitters from the processing of photonic circuits, facilitating post‐fabrication assembly, modularity, and improved device yields—factors that are all essential for scaling toward functional Q‐PICs.

**FIGURE 7:**
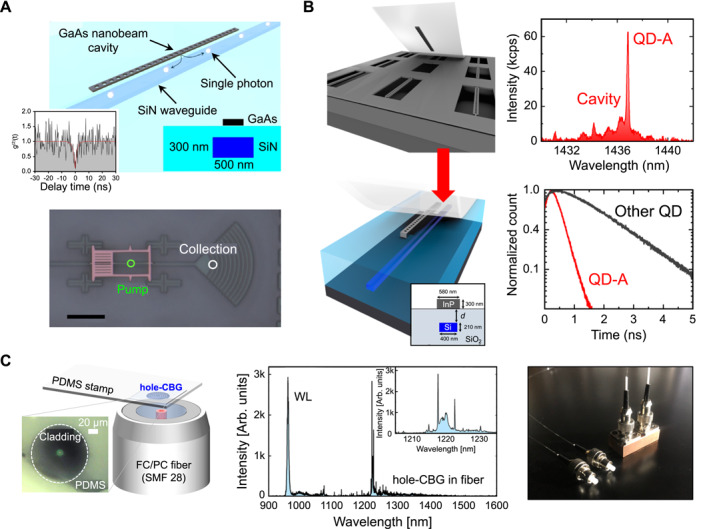
Integration of III–V quantum dot single‐photon sources with functional photonic structures through PDMS‐based transfer‐printing. (A) InAs/GaAs QDs embedded in a one‐dimensional PhC cavity transfer‐printed onto a SiN waveguide. The guided single‐photon emission is validated with Purcell‐enhanced decay dynamics and clear photon antibunching. (B) Telecom‐band InAs/InP QD integrated into a nanobeam PhC cavity and transfer‐printed onto a CMOS‐processed Si photonic chip. The device demonstrates spectrally matched cavity–emitter coupling, a six‐fold lifetime reduction, and high‐efficiency waveguide coupling. (C) Plug‐and‐play fiber‐coupled SPS formed by transfer‐printing a hole‐CBG with an embedded InAs/InP QD directly onto the core of a single‐mode fiber. The resulting all‐fiber device enables efficient single‐photon delivery through a standard fiber channel with long‐term operational stability. Reproduced with permission from Ref. [178], Optica Publishing Group (A), Ref. [179], IOP Publishing (B), and Ref. [180], Wiley Online Library (C).

An approach in which InAs/GaAs QDs embedded in one‐dimensional phC nanobeam cavities are integrated onto SiN waveguides through transfer‐printing is shown in Figure 7A. By leveraging the second‐order cavity mode in momentum space, the researchers achieved phase matching with the low‐refractive index SiN waveguide, effectively addressing the intrinsic index mismatch between GaAs (*n* = 3.4) and SiN (*n* = 2.0). The resulting device demonstrated a Purcell factor of approximately 4, a total QD‐to‐waveguide coupling efficiency of ∼53%, and confirmed single‐photon emission, as evidenced by a second‐order correlation of $g^{\left(2\right)}\left(0\right)$ = 0.10. The use of PDMS transfer‐printing has been pivotal in achieving precise positioning of the suspended GaAs nanobeam on the pre‐fabricated SiN platform, a task that poses significant challenges when employing conventional direct growth or bonding methods owing to issues related to lattice mismatch and thermal expansion. This research underscores the potential for efficient emitter–waveguide coupling on low‐refractive‐index SiN platforms, particularly when mode engineering is integrated with a high‐precision nondestructive transfer process.

Building on these methodologies, the integration of QDs with PICs has significantly broadened the horizons for scalable quantum technologies [43, 175, 176, 179, 181]. The hybrid integration of InAs/InP QDs operating at 1.44 μm into InP PhC nanobeam cavities, which are transfer‐printed onto CMOS‐processed Si photonic chips, is shown in Figure 7B. Time‐resolved photoluminescence measurements revealed a six‐fold reduction in lifetime, corresponding to a Purcell factor of approximately 6, alongside a waveguide‐coupling efficiency of approximately 82%. Single‐photon purity was validated with $g^{\left(2\right)}\left(0\right)\approx 0.20$. Notably, this integration extended to fiber‐pigtailed Si chips, facilitating direct single‐photon emission through optical fibers. Here, PDMS transfer‐printing has been instrumental in backend integration, enabling the combination of fully processed CMOS Si photonic chips with pre‐characterized QD devices without compromising the integrity of the foundry‐fabricated circuits. The pick‐and‐place capability maintains the integrity of CMOS workflows and supports modular packaging strategies, allowing for the selective addition or replacement of emitters to optimize overall performance [43].

The direct integration of QDs with optical fibers presents a promising avenue for advancing practical quantum communication systems [180, 182]. As shown in Figure 7C, a “plug‐and‐play” architecture has been developed by transfer‐printing QD devices based on hole‐type circular Bragg gratings (h‐CBGs) directly onto the core of standard single‐mode fibers. This innovative approach facilitates the direct emission of single photons into the fiber, eliminating the need for free‐space alignment. The fiber‐integrated SPS demonstrated a Purcell factor of approximately 4, with collection efficiencies ranging from 24% to 30% at the first lens, and a measured QD‐to‐fiber efficiency of 8.1%. Single‐photon emission was validated with a $g^{\left(2\right)}\left(0\right)$ value of approximately 0.16. Although the fiber‐coupling efficiency is currently lower than that of chip‐based approaches, the plug‐and‐play design significantly enhances the practicality and long‐term stability of field‐deployable quantum light sources. Notably, the use of PDMS micro transfer‐printing enabled precise alignment of the h‐CBG cavity with the fiber core within an accuracy of less than 500 nm, ensuring adequate mode overlap. Without such a high‐precision, cost‐effective transfer method, reliable and scalable assembly of fiber‐integrated QD devices would be unfeasible. Collectively, these advancements underscore the flexibility of PDMS transfer‐printing for integrating QD SPSs across heterogeneous photonic platforms. Transfer‐printing maintains emitter quality while enabling post‐fabrication placement flexibility across low‐index dielectric waveguides, CMOS Si platforms, and direct integration with optical fibers. Achievements, such as Purcell enhancement, on‐chip coupling efficiencies reaching up to 82%, and fiber‐based plug‐and‐play operation, represent significant advancements toward scalable and reliable quantum light sources. Beyond simple alignment, PDMS transfer‐printing is instrumental in achieving the precise integration of nanoscale dipole emitters within complex quantum photonic circuits. Anticipated advancements include deterministic spectral matching, site‐controlled QDs, and innovative packaging strategies that address the efficiency gap between chip‐based and fiber‐based devices, enabling large‐scale deployment of QD‐based quantum photonic systems.

### 4.2 Quantum Light Sources Based on 2D Semiconductors

**FIGURE 8:**
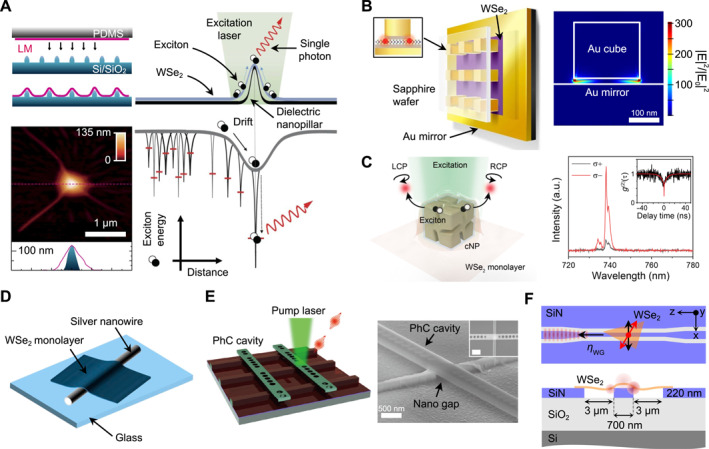
PDMS transfer‐printing for strain‐engineered 2D single‐photon sources and self‐aligned photonic integration. (A) Deterministic strain‐induced SPSs formed through PDMS monolayer TMD transfer‐printing over nanopillars, resulting in exciton funneling and single‐photon emission. (B) Ag nanocube–Au mirror gap cavity whose sharp corners co‐localize strain and optical hot spots, enabling Purcell‐enhanced single‐photon emission from the same site. (C) Chiral gold nanoparticle with multiple nanogaps that seeds local strain and imparts circular polarization, resulting in SAM‐encoded single photons without magnetic fields. (D) Monolayer positioned on an Ag nanowire, where contact‐line strain self‐aligns emitters to the SPP mode for efficient plasmonic coupling. (E) Anisotropic‐strain nanogap/rod template that sets a linear dipole, transfer‐printed onto a 1D PhC cavity for polarization‐matched coupling and Purcell enhancement. (F) WSe_2_ monolayer transfer‐printed onto a CMOS‐compatible SiN chip for the direct coupling of strain‐induced emitters into guided TE modes. Reproduced with permission from Refs. [183, 184, 193, 194], Nature Publishing Group (A), (B), (F), Ref. [195], American Association for the Advancement of Science (C), and Refs. [196, 197] American Chemical Society (D), (E).

Two‐dimensional (2D) semiconductors offer robust, bright, and spectrally narrow single‐photon emitters (SPEs). A primary challenge lies in the deterministic placement of these emitters and their seamless integration with on‐chip photonic structures, all while maintaining high optical quality. Micro transfer‐printing using PDMS presents a versatile solution, enabling the deterministic generation of quantum emitter arrays at predefined locations and facilitating the self‐alignment of these emitters with optical micro‐ and nanostructures for photonic multifunctionality. In this approach, monolayer 2D semiconductors are precisely positioned over topographic nanoscale templates through the micro transfer‐printing method [183, 184, 185, 186, 187, 188, 189, 190, 191, 192]. As shown in Figure 8A, nanopillar structures induce localized tensile strain in the monolayer semiconductors, effectively funneling excitons and generating deterministic SPEs. Nanopillar arrays can support hundreds of strained sites per monolayer flakes (spanning tens of microns), achieving near‐unity SPE generation yields and nanometer‐scale placement accuracy.

Expanding upon the strain–template approach, micro transfer‐printing assumes an additional function when the nanoscale template itself serves as a functional photonic structure. The engineered topography of these photonic templates not only induces localized strain but also facilitates self‐alignment of nascent SPS to the optical modes. This is particularly advantageous for plasmonic resonances, where the effective mode volume is deeply subwavelength and the system demonstrates low alignment tolerance [193, 195, 198, 199]. For example, in the Ag‐nanocube/Au‐mirror gap cavity (Figure 8B), the corners of the nanocube simultaneously define the strain trap and field hot spot, enabling deterministic generation of emitters precisely at the cavity maximum. The broadband (low‐Q) nature of the cavity ensures spectral matching between the coupled emitters and cavity mode, resulting in Purcell factors up to approximately 551 (approximately 181 on average), single‐photon rates up to approximately 42 MHz, and linewidths near approximately 55 μeV. In a complementary plasmonic platform, chiral gold nanoparticles with multiple nanogaps (Figure 8C) are employed to both seed local strain in monolayer WSe_2_ and imprint strong circular polarization to the emitted photons—all within a subwavelength footprint and with no external magnetic fields. Emitters deterministically generated at the nanoparticle sites demonstrate pronounced antibunching ($g^{\left(2\right)}\left(0\right)=0.3$) and a significant degree of circular polarization, demonstrating spin‐angular‐momentum–encoded single‐photon generation through near‐field chiral optics. At the plasmonic waveguiding limit, conformally draping a monolayer over colloidal Ag NWs generates a deterministic strain gradient along the contact line, ensuring that the emitter forms within the SPP near field (Figure 8D) [196]. The curvature and contact line generate a deterministic strain gradient, enabling direct coupling emission from the SPS into the propagating plasmon. A lower‐bound coupling efficiency of approximately 26% into the NW mode has been demonstrated, with remote readout achievable at the wire terminus. Although achieving a sub‐10‐nm field confinement in plasmonic modes necessitates near‐perfect spatial overlap, this self‐alignment enables exceptionally large Purcell enhancements and robust photon routing, even in the presence of metallic losses.

In contrast, dielectric nanophotonics offers low optical loss, high‐Q resonances, phase stability, and compatibility with foundry‐scale fabrication, supporting quantum photonic applications through cavity quantum electrodynamics and Q‐PICs. However, unlike plasmonic hot spots that are localized at the nanotemplate surface, dielectric modes are predominantly confined within high‐index media. Consequently, surface‐resident 2D SPSs, despite precise in‐plane alignment achieved through transfer‐printing, are positioned only at the evanescent tail of the optical mode. To address this challenge, various strategies have been developed to efficiently couple 2D SPS, which are inevitably located on the surface of nanotemplates, with dielectric optical modes. As shown in Figure 8E, engineered anisotropic tensile strain using a dielectric nanogap or rod structure can define the linear polarization of the emission [197]. By transfer‐printing a pre‐fabricated cavity onto a strained monolayer, the polarization of the emitter can be aligned with a cavity eigenmode of 1D PhC. This approach provides a practical route to polarization‐matched, high‐Q cavity coupling, enabling Purcell‐enhanced extraction and improved indistinguishability through cavity filtering. For scalable Q‐PICs, as discussed in Section 4.1, the integration of 2D SPS with CMOS‐compatible SiN waveguide has been achieved through transfer‐printing onto pre‐fabricated waveguides (Figure 8F) [194]. Two complementary levers enhanced field overlap and optical coupling efficiency. First, slot waveguides confined the electric field within a low‐index gap that is accessible to the 2D monolayer, thereby increasing the spatial overlap between the SPS and guided transverse electric (TE) mode and resulting in improved coupling efficiency. Second, cavity–waveguide co‐design directs emission into the waveguide modes with controllable external coupling, driving the β‐factor and waveguide extraction toward unity. Through the combined use of optical dipole orientation control and spatial mode engineering, emitter–cavity–waveguide systems can achieve significantly stronger evanescent coupling compared with bulk configurations. These advances establish a foundation for future design efforts focused on optimizing single‐photon extraction and enhancing the indistinguishability of photons coupled into the guided waveguide mode.

## 5 Current Challenges and Future Outlook

This review has highlighted recent advances in PDMS‐based micro‐transfer printing as a versatile and scalable integration paradigm for wavelength‐scale light sources. These developments indicate that transfer‐enabled integration has reached a level where diverse nano‐ and quantum materials, resonant nanostructures, and integration schemes can be combined on‐demand within a single device; however, several critical challenges must still be resolved before this technology can be adopted as a truly practical and industry‐ready solution. In this section, we discuss three major bottlenecks that current technology must overcome and outline potential solutions together with future perspectives.

*Accuracy and alignment*: Despite the successful demonstrations in achieving sub‐micrometer alignment of the aforementioned classes of light sources on pre‐patterned Si and Si3N4 waveguides, the tolerances demanded by next‐generation high‐density PICs remain extremely challenging. Efficient evanescent or butt coupling to wavelength‐scale cavities typically requires lateral and vertical misalignments well below a few hundred nanometers [200, 201, 202], particularly for PhC nanobeams and NW‐waveguide junctions, where the modal overlap and scattering loss are highly sensitive to the relative position and orientation of the active emitter and passive waveguide [41, 67, 203]. In many of the demonstrations in this review, the integration accuracy is sufficient to prove the underlying concept, but there is still a clear gap between such proof‐of‐principle alignment and the deterministic, wafer‐scale placement accuracy required for complex PICs with high density routers and multi‐channel couplers [34, 201, 204]. Moreover, three‐dimensional integration schemes inevitably introduce additional alignment degrees of freedom in the out‐of‐plane direction, further complicating process control [103, 205, 206, 207].

Future work must therefore focus on closing this accuracy gap by combining transfer‐printing with advanced alignment and registration strategies. Promising avenues include the use of high‐precision alignment marks that can be recognized by machine–vision systems, closed‐loop feedback control of stamp position [87, 205, 208, 209] and kinetic movement during the contact and peel‐off [105, 210, 211, 212], and hybrid schemes that leverage self‐aligned cavity or waveguide designs whose coupling efficiency is less sensitive to positional errors [41, 167]. In parallel, design concepts that decouple the optical cavity from the gain medium offer an attractive route to relax alignment tolerances, because the modal confinement is primarily defined by the pre‐fabricated resonators [69, 202, 213, 214].

*Throughput, automation, and scalability*: A central bottleneck for the technology stems from the trade‐off between deterministic placement and process throughput. As discussed, conventional techniques (e.g., wafer‐ or die‐bonding) providing high throughput and the potential for wafer‐scale processing sacrifice local on‐demand placement and consume large areas of epitaxial III–V material. In contrast, PDMS‐based micro‐transfer‐printing enables selective integration of pre‐screened devices with high positional accuracy [71, 72, 215]. However, it currently operates in a kinetically controlled serial fashion, which limits the number of devices that can be assembled within a practical processing time. This constraint is particularly critical for applications that demand large arrays of independent lasers or emitters, such as wavelength‐division‐multiplexed transceivers, neuromorphic photonic processors, and multi‐channel quantum photonic circuits, where hundreds of thousands of individually addressable light sources may be required on a single chip.

To overcome this bottleneck, the development of parallelized and automated transfer‐printing systems is essential. One simple approach is to engineer multi‐post or arrayed micro‐stamp architectures [36, 106, 116] that can pick up and place many devices in a single cycle while preserving selectivity and avoiding damage of neighboring structures [72, 113]. Another approach is to integrate robotics [208, 216], machine–vision algorithms [33, 208, 217] and power‐controlled laser systems [72, 215] that can autonomously identify target emitters, evaluate their optical performance (e.g., rapid PL screening), and execute rapid and programmable pick‐and‐place operations [133, 216, 218], enabling both localized and large‐area transfers with minimal human intervention. Ultimately, bridging the gap between single‐device laboratory‐scale demonstrations and industrial manufacturing will require co‐optimization of stamp materials and geometries, motion‐control hardware, and process recipes to achieve both high throughput and high yield across full wafers or large panels.

*Reproducibility, reliability, and long‐term stability*: The reproducibility and reliability of transfer‐integrated light sources are another primary concern for their deployment in commercial photonic systems. More specifically, systematic studies of device‐to‐device variability, long‐term aging, and dominant failure mechanisms must be carried out on statistically meaningful populations of transfer‐integrated devices [67, 208, 215]. The transfer process itself can introduce variability through factors such as local stamp deformation [219, 220], subtle differences in adhesive forces [105, 211, 221] and microscale stresses generated during the contact and peeling steps [108, 210, 211, 222], which may influence cavity Q‐factors, emission wavelengths, and coupling efficiencies.

Addressing these issues calls for advances at both materials and process levels. At the materials level, there is a need to develop stamp formulations or surface modifications with tunable viscoelastic properties [37, 107, 223], controllable surface chemistry [131, 132], and improved environmental stability [126, 224, 225], such that adhesion can be modulated precisely without leaving residues or inducing damage to delicate nanostructures. For example, functional materials such as shape‐memory polymers with microstructured surfaces that enable temperature‐controlled adhesion [226, 227] can be integrated with various stamp architectures to facilitate reproducible and reliable transfer‐integration. At the process level, the adoption of standardized operating procedures for contact time and peel speed, and environmental conditions (e.g., temperature, humidity, etc.), together with monitoring of optical performance, will be crucial to ensure reproducibility from batch to batch. Long‐term reliability studies, including accelerated aging tests under harsh environmental conditions, high duty‐cycle optical pumping, and continuous current injections, will provide essential data to identify dominant degradation pathways and to guide the optimal design for robust device architectures.

*Future outlook*: Looking ahead, micro‐transfer‐enabled integration of wavelength‐scale light sources is prepared to play a central role in accelerating the photonic and quantum technologies, provided that the aforementioned challenges can be systematically overcome. In the near term, the most impactful developments are expected to combine process engineering with photonic design. For example, a laser‐combined automated transfer system enabling machine–vision‐assisted alignment will operate the process with an engineered stamp architecture to perform parallel device manipulation, whereas cavities and waveguides will be designed to be intrinsically tolerant to residual alignment errors and mechanical perturbations. In the long term, the same capabilities that make transfer‐printing attractive for classical photonics are likely to become indispensable for quantum photonic systems. The site‐controlled photon emitter array and deterministic assembly of wavelength‐scale lasers, and nonlinear elements onto low‐loss PICs coupled with highly sensitive photon detectors, will form the basis of scalable quantum networks, photonic quantum processors, and on‐chip quantum light sources. Realizing this vision will require close collaboration across material science, device physics, mechanics, and system engineering, as well as the establishment of standardized CMOS‐compatible process flows for micro‐transfer‐enabled photonic integration.

## Author Contributions

J.‐P.S., M.K.K. and Y.‐S.N. initiated the review, defined its scope, organized the paper sections, and supervised the project. H.K. and D.S. wrote the manuscript. G.Y.B., G.‐W.L. and H.Y.J. prepared and refined the figures and schematics. Y.‐S.N. and H.K. reviewed and edited the manuscript. All authors have accepted responsibility for the entire content of this manuscript and consented to its submission to the journal, reviewed all the results and approved the final version of the manuscript.

## Funding

This study was supported by the National Research Foundation of Korea (NRF) grant funded by the Korea government (MSIT) (No. RS‐2024‐00343969, RS‐2025‐00558607, RS‐2025‐25445839 RS‐2025‐25460412, RS‐2025‐25465325), and funded by the Ministry of Education (No. RS‐2021‐NR060140, RS‐2025‐25441317), Institute for Information and Communications Technology Planning and Evaluation (IITP) grants (2025‐RS‐2022‐00164799, 2022‐0‐00198, RS‐2025‐25464252), and KIST Institutional Program (2E33571). This paper was written as part of Konkuk University's research support program for its faculty on sabbatical leave in 2024.

## Conflicts of Interest

The authors declare no conflicts of interest.

## Data Availability

Data sharing is not applicable to this article as no datasets were generated or analyzed during the current study.
